# Supplementary material for: From sequence to enzyme mechanism using multi-label machine learning
Source: BMC Bioinformatics. 2014 May 19;15:150. doi: 10.1186/1471-2105-15-150 (PMC4229970; doi:10.1186/1471-2105-15-150)
Supplement: Additional file 2 — Java code of ml2db. Additional file ml2db_code.tar.gz contains the Java source code to run the multi-label machine learning experiments and save the results to database. The code’s Javadoc is included. [file 1471-2105-15-150-S2.zip › additional file 2/ml2db/ml2db/doc/test/ml2db/dataharness/DataTwo.html]

DataTwo


JavaScript is disabled on your browser.


- Overview
- Package
- Class
- Use
- Tree
- Deprecated
- Index
- Help

- Prev Class
- Next Class

- Frames
- No Frames

- All Classes

- Summary:
- Nested |
- Field |
- Constr |
- Method

- Detail:
- Field |
- Constr |
- Method


test.ml2db.dataharness

## Class DataTwo

- java.lang.Object
- - test.ml2db.dataharness.DataTwo

- ---

    

  ```
  public class DataTwo
  extends java.lang.Object
  ```

  Class

  Version:
  :   25 Feb 2011

  Author:
  :   Luna De Ferrari ldf(at)st-andrews.ac.uk

- - ### Field Summary

    Fields

    | Modifier and Type | Field and Description |
    | --- | --- |
    | `static java.lang.String` | `arffEmptyInstance` |
    | `static java.lang.String` | `arffInstance1` |
    | `static java.lang.String` | `arffInstance1b` |
    | `static java.lang.String` | `arffInstance2` |
    | `static java.lang.String` | `arffInstance4` |
    | `static java.lang.String` | `arffInstanceAttVoid` |
    | `static java.lang.String` | `arffInstanceClassVoid` |
    | `static java.lang.String` | `DATASET_NAME` |
    | `static java.lang.String` | `INST1` |
    | `static java.lang.String` | `INST2` |
    | `static java.lang.String` | `INST3` |
    | `static java.lang.String` | `INST4` |
    | `static int` | `NUMBER_OF_INSTANCES_IN_TEST_ARFF` |
  - ### Constructor Summary

    Constructors

    Constructor and Description

    `DataTwo()`
  - ### Method Summary

    Methods

    | Modifier and Type | Method and Description |
    | --- | --- |
    | `static uk.ac.standrews.utils.main.maputils.OneToManyMap<java.lang.String,java.lang.String>` | `getInstanceAttributeMap()` |
    | `static uk.ac.standrews.utils.main.maputils.OneToManyMap<java.lang.String,java.lang.String>` | `getInstanceClassMap()` |

    - ### Methods inherited from class java.lang.Object

      `equals, getClass, hashCode, notify, notifyAll, toString, wait, wait, wait`

- - ### Field Detail


    - #### NUMBER\_OF\_INSTANCES\_IN\_TEST\_ARFF

      ```
      public static int NUMBER_OF_INSTANCES_IN_TEST_ARFF
      ```


    - #### arffInstance1

      ```
      public static final java.lang.String arffInstance1
      ```

      See Also:
      :   Constant Field Values


    - #### arffInstance1b

      ```
      public static final java.lang.String arffInstance1b
      ```

      See Also:
      :   Constant Field Values


    - #### arffInstance2

      ```
      public static final java.lang.String arffInstance2
      ```

      See Also:
      :   Constant Field Values


    - #### arffInstance4

      ```
      public static final java.lang.String arffInstance4
      ```

      See Also:
      :   Constant Field Values


    - #### arffEmptyInstance

      ```
      public static final java.lang.String arffEmptyInstance
      ```

      See Also:
      :   Constant Field Values


    - #### arffInstanceClassVoid

      ```
      public static final java.lang.String arffInstanceClassVoid
      ```

      See Also:
      :   Constant Field Values


    - #### arffInstanceAttVoid

      ```
      public static final java.lang.String arffInstanceAttVoid
      ```

      See Also:
      :   Constant Field Values


    - #### INST1

      ```
      public static final java.lang.String INST1
      ```

      See Also:
      :   Constant Field Values


    - #### INST2

      ```
      public static final java.lang.String INST2
      ```

      See Also:
      :   Constant Field Values


    - #### INST3

      ```
      public static final java.lang.String INST3
      ```

      See Also:
      :   Constant Field Values


    - #### INST4

      ```
      public static final java.lang.String INST4
      ```

      See Also:
      :   Constant Field Values


    - #### DATASET\_NAME

      ```
      public static final java.lang.String DATASET_NAME
      ```

      See Also:
      :   Constant Field Values
  - ### Constructor Detail


    - #### DataTwo

      ```
      public DataTwo()
      ```
  - ### Method Detail


    - #### getInstanceAttributeMap

      ```
      public static uk.ac.standrews.utils.main.maputils.OneToManyMap<java.lang.String,java.lang.String> getInstanceAttributeMap()
      ```


    - #### getInstanceClassMap

      ```
      public static uk.ac.standrews.utils.main.maputils.OneToManyMap<java.lang.String,java.lang.String> getInstanceClassMap()
      ```


- Overview
- Package
- Class
- Use
- Tree
- Deprecated
- Index
- Help

- Prev Class
- Next Class

- Frames
- No Frames

- All Classes

- Summary:
- Nested |
- Field |
- Constr |
- Method

- Detail:
- Field |
- Constr |
- Method
